# Protective Effects of Angiotensin Receptor Blockers on the Incidence of Dementia in Patients with Chronic Kidney Disease: A Population-Based Nationwide Study

**DOI:** 10.3390/jcm10215175

**Published:** 2021-11-05

**Authors:** Yi-Hsien Chen, Yun-Yi Chen, Yu-Wei Fang, Ming-Hsien Tsai

**Affiliations:** 1 Division of General Medicine, Department of Internal Medicine, Shin-Kong Wu Ho-Su Memorial Hospital, Taipei 11101, Taiwan; iangarfcloud@gmail.com; 2 Department of Research, Shin-Kong Wu Ho-Su Memorial Hospital, Taipei 11101, Taiwan; yunyichen@gmail.com; 3 Institute of Hospital and Health Care Administration, National Yang Ming Chiao Tung University, Taipei 112304, Taiwan; 4 Division of Nephrology, Department of Internal Medicine, Shin-Kong Wu Ho-Su Memorial Hospital, Taipei 11101, Taiwan; m005916@gmail.com; 5 Department of Medicine, Fu-Jen Catholic University School of Medicine, Taipei 242062, Taiwan

**Keywords:** chronic kidney disease, National Health Insurance Research Database, angiotensin receptor blockers, dementia

## Abstract

Chronic kidney disease (CKD) is associated with dementia. Angiotensin receptor blockers (ARBs) have been widely used for delaying CKD progression; however, their effect on dementia prevention in patients with CKD remains unclear. We designed a retrospective cohort study to investigate the effects of ARBs on the incidence of dementia in patients with CKD. We selected 21,208 patients from the Taiwan nationwide database from 1 January 2006 to 31 December 2006. We identified ARB users (*n* = 17,466) and ARB non-users (*n* = 3742) and their medication possession ratio (MPR). The Cox proportional hazard model was used to estimate hazard ratios (HRs) for the incidence of dementia in ARB users in the CKD population. During the 11-year follow-up period, 2207 dementia events were recorded; multivariate-adjusted hazard ratios for dementia by ARB usage and ARB usage per MPR were 0.578 (95% CI: 0.52–0.643) and 0.996 (95% CI: 0.995–0.998), respectively. This association was observed in almost all subgroups. Dose frequency effect of ARBs was noted; patients with higher MPRs of ARBs generally had higher protection from dementia. Patients with hypertension and CKD who received ARBs had a decreased risk of dementia. Protective effects of ARBs on dementia increased with the frequency of ARB use.

## 1. Introduction

Chronic kidney disease (CKD), a major health problem that causes considerable financial burden worldwide, can lead to end-stage renal disease (ESRD), resulting in the requirement of renal replacement therapy and contributing to increased cardiovascular mortality and morbidity [1,2]. The estimated global prevalence of CKD is 13.4% [3], with Taiwan having a higher prevalence of CKD (11.9–15.5%) [4,5]. Moreover, studies have reported that CKD is an independent risk factor for cognitive impairment and dementia [6,7], which are major health concerns in the older population worldwide [8]. Both the kidneys and brain are highly vascular structures and thus susceptible to vascular injuries. Therefore, patients with CKD might develop vascular dementia [9]. CKD may lead to cognitive impairment through various mechanisms. First, patients with CKD have a high prevalence of risk factors for cardiovascular disease (CVD); these are also risk factors for cerebrovascular disease. Second, patients with CKD are prone to develop clinical cerebrovascular diseases such as stroke, transient ischemic attack, and lacunar infarction. Third, uremic toxins might worsen the cognitive deficit of patients with CKD [10]. However, the underlying pathophysiology is still not well understood.

CKD is commonly associated with hypertension as a comorbidity. Angiotensin-converting enzyme inhibitors (ACEIs) and angiotensin receptor inhibitors (ARBs) are the preferred agents for treating hypertension in the CKD cohort due to their ability to delay the progression of CKD [11,12]. The modulation of the renin–angiotensin system (RAS) in the brain through ACEIs or ARBs may exert neuroprotective effects [13]. ARBs can reduce the risk of Alzheimer’s disease and dementia [14,15,16,17]. However, two recent meta-analyses have reported that antihypertensive therapy could reduce the risk of dementia; this finding might be attributable to the decline in blood pressure but not the class effects of any antihypertensive drug [18,19]. Therefore, apart from blood pressure reduction, the efficacy of RAS blockade in dementia prevention remains unclear.

ACEIs are associated with cough and a considerably but relatively low angioedema risk; the overall withdrawal rate due to adverse events is higher for ACEIs than ARBs [20]. ACEIs caused cough in up to 35% of Chinese patients [21]. Because ARBs are widely used as the antihypertensive agent in patients with CKD in the Chinese population, if there is any association between the use of ARBs and the development of dementia remains unclear. 

In the present study, we examined the correlation between the usage of ARBs and the incidence of dementia in the CKD population by using real-world data from the National Health Insurance (NHI) Research Database (NHIRD).

## 2. Materials and Methods

### 2.1. Data Source and Research Samples

The present study used CKD thematic data from the NHIRD maintained by the Health and Welfare Data Science Center (HWDC), Ministry of Health and Welfare (MOHW), Taiwan. CKD was defined as having 3 outpatient diagnoses, with an interval of more than 4 weeks between each other within 1 year; the diagnosis of CKD includes 124 ICD-9-CM codes that were verified officially in the Chronic Kidney Disease Prevention Technology Research Project conducted by the Health Promotion Administration, Ministry of Health and Welfare. This includes CKD stages from 1 to 5 (Supplemental Table S1). The NHIRD includes the health-care utilization records of 99.9% of Taiwan’s 23 million population enrolled in Taiwan’s NHI since its launch in 1995 [22]. The NHIRD contains information regarding patients’ birth date, sex, residential area, diagnostic codes, medical procedures, and drug prescriptions. In the NHIRD, disease diagnoses are defined according to the International Classification of Diseases, Ninth Revision, Clinical Modification (ICD-9-CM) codes before the year 2015 and International Classification of Diseases, Tenth Revision, Clinical Modification (ICD-10-CM) codes after the year 2015. Before the NHIRD data are released for the research purpose, the personal information of all beneficiaries is deidentified and anonymized to ensure patients’ privacy following rigorous secrecy guidelines. Therefore, the requirement of informed consent was waived by the Ethics Review Board of Shin-Kong Wu Ho-Su Memorial Hospital (IRB approval number: No. 20200806R).

### 2.2. Study Design and Study Population 

**Figure 1:**
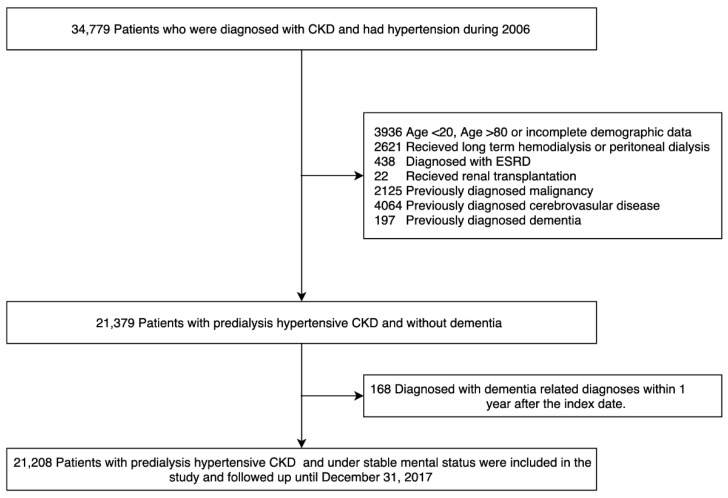
Schema of patient enrollment in the study.

This was a population-based longitudinal cohort study. From the NHIRD, we identified and recruited patients diagnosed as having CKD from 1 January 2006, to 31 December 2006 (*n* = 34,779). Patients who were previously diagnosed as having cancer, ESRD, dementia, or cerebrovascular disease; were receiving regular hemodialysis or peritoneal dialysis; had previously undergone renal transplantation; were aged <18 or >80 years; and had missing information were excluded. Finally, a total 21 379 patients with CKD and hypertension were included in this study (Figure 1). The index date was defined as 1 January 2007. 

Baseline comorbidities, namely ischemic heart disease, hyperlipidemia, diabetes, atrial fibrillation, chronic heart failure, asthma, chronic obstructive pulmonary disease, peripheral artery occlusion disease, major depression, Parkinson’s disease, rheumatic arthritis, thyrotoxicosis, hypothyroidism, gout, and insomnia, were considered when the patients had at least three outpatient diagnoses or one inpatient diagnosis within 1 year before the index date (Supplemental Table S2). Usage of other drugs, namely aspirin, benzodiazepines, anticoagulants, nonsteroidal anti-inflammatory drugs (NSAIDs), acetaminophen, insulin, statins, calcium channel blockers, metformin, beta-blockers, and antiplatelets, was considered when the patients used the drug for at least 3 months within 1 year before the index date (Supplemental Table S3).

### 2.3. Definition of Angiotensin Receptor Inhibitor (ARB) Usage

We calculated the medication possession ratio (MPR) to estimate the degree to which the patients were prescribed ARBs during the follow-up period. The MPR is calculated as the sum of the days on which the drug was used during the observation period, divided by the total number of days in the observation period [23]. In addition, we defined ARB non-users and users as those with an MPR of 0% and >0%, respectively.

### 2.4. Definition of Dementia

The primary outcome of this study was the diagnosis of dementia, which was determined based on whether a patient was eligible for the NHI Administration IC Card for Severe Illness issued by the National Health Insurance Administration for dementia-related diagnoses. The data were analyzed from the index date (1 January 2007) to the first diagnosis of dementia, as defined previously, or the end of the study period (31 December 2017). This study used the criteria of the application for the NHI Administration IC Card for Severe Illness for dementia (Item 6-1, ICD-9-CM 290 or ICD-10-CM F01.50, F01.51, F03.90, and F03.91 diagnosed by a licensed neurologist or psychiatrist).

### 2.5. Statistical Analysis

Continuous variables are expressed as means ± standard deviations, whereas categorical variables are expressed as counts with proportions. Differences in the variables between the groups (ARB users vs nonusers) were determined using the chi-squared test for the proportions of continuous variables and the *t* test for the means of continuous variables. The Kaplan–Meier method was used to estimate and plot dementia-free curves, which were evaluated using the log-rank test. A Cox proportional regression model was used to estimate hazard ratios (HRs) and 95% CIs for the risk of dementia. Both crude and multivariable variables were adjusted. The assumption of the proportional hazard was not violated when the interaction between time and the variables was examined. Moreover, we considered the death as a competing risk of dementia diagnosis in our analysis.

All statistical analyses were performed using SAS, version 9.4 (SAS Institute, Cary, NC, USA). For all tests, a two-sided *p* value of < 0.05 was considered statistically significant.

### 2.6. Sensitivity Analysis

To examine the reliability of our findings, we used a separate regression model for modeling dichotomic ARB usage (yes vs no) or the MPR of ARBs as a function of the dementia event by using a modified stepwise procedure by using 4 modeling steps; the variables of demographic data, comorbidities, and medications were added into the models step by step. In addition, we analyzed the MPR of ARBs during the follow-up period to investigate the dose frequency effect of ARBs on the prevention of dementia. The Cochran–Armitage test was performed to examine the dose frequency effect of ARBs. We stratified the ARB users by the MPR and included them in the following groups: those with an MPR of ≤20%, 20%, 30%, 40%, 50%, 60%, 70%, 80%, and >80%. We considered the group with an MPR of ≤20% as our reference point. Finally, we performed a subgroup analysis by age, sex, and comorbidities to test the consistency of the protective effect of ARBs on dementia in patients with CKD.

## 3. Results

### 3.1. Patient Characteristics

We enrolled 21,208 patients with hypertension and CKD in the present study (Table 1). The mean age of the enrolled patients was 61.09 ± 12.10 years, and 56.89% and 56.01% of the patients were women and had diabetes, respectively. Among them, 17,466 patients were ARB users, and the others were ARB nonusers. 

Table 1 lists differences in baseline characteristics between the ARB users and nonusers. Female patients; younger patients, patients with comorbidities, namely hyperlipidemia, diabetes, and congestive heart failure, and patients without atrial fibrillation and Parkinson’s disease were more likely to be prescribed ARBs (all *p* < 0.05). Moreover, the ARB users were more likely to be prescribed antiplatelet agents, insulin, statin, metformin, ACEIs, calcium channel blockers, and beta-blocker, whereas the ARB nonusers were more likely to be prescribed acetaminophen and benzodiazepine (all *p* < 0.05).

### 3.2. Risk Factors for Dementia in our Hypertensive CKD Cohort

A total of 2207 dementia events were noted during the 11-year observation period. The incidence rates of dementia in the ARB users and nonusers were 0.011 and 0.016 per person-year (PY), respectively. Table 2 lists possible risk factors for dementia. Female sex; older age; comorbidities, namely ischemic heart disease, diabetes mellitus, atrial fibrillation, chronic heart failure, peripheral arterial disease, asthma, chronic obstructive pulmonary disease (COPD), major depressive disorder, Parkinson’s disease, rheumatoid arthritis, and insomnia; and use of antiplatelet agents, benzodiazepines, anticoagulants, NSAIDs, acetaminophen, insulin, calcium channel blockers, diuretics, and metformin were determined to be significant risk factors for dementia. 

After adjusting for multivariable factors, only female sex; comorbidities, namely ischemic heart disease, diabetes mellitus, chronic heart failure, COPD, major depressive disorder, Parkinson’s disease, and insomnia; and the usage of antiplatelet agents, benzodiazepines, insulin, diuretics, and metformin were found to be significant risk factors for dementia. Significant protective effects of ARBs on the incidence of dementia were observed in both crude and adjusted analyses. Although we observed significant protective effects of ACEIs on the incidence of dementia, the results of crude analysis were not significant.

### 3.3. Effects of ARB Usage on the Incidence of Dementia

**Figure 2:**
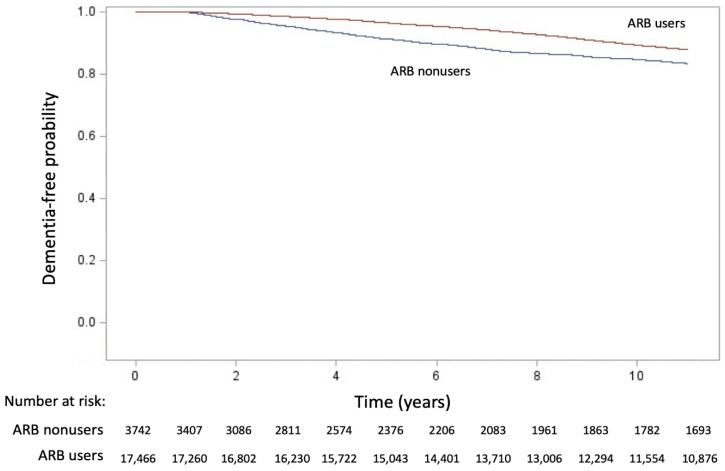
Kaplan–Meier cumulative event-free plots of dementia. A significant difference in entering dialysis was noted between groups (log-rank test, χ^2^ = 68.56, *p* < 0.001).

The results of the Kaplan–Meier analysis revealed that a significant difference in dementia-event-free curves between the ARB users and non-users (χ^2^ = 68.56, *p* < 0.001; Figure 2). The protective effect of ARBs on the incidence of dementia was observed in all the models (Table 3). The effects of ARB usage (yes vs. no) were investigated using four stepwise variable–adjusted models; the results were consistent in all the models (HR: 0.645, 95% CI: 0.581–0.716; HR: 0.607, 95% CI: 0.547–0.674; HR: 0.589, 95% CI: 0.530–0.655; and HR: 0.578, 95% CI: 0.520–0.643, respectively). Moreover, the risk of dementia significantly decreased with an increase in the MPR of ARBs in the four regression models (HR: 0.997, 95% CI: 0.996–0.998; HR: 0.997, 95% CI: 0.996–0.998; HR: 0.997, 95% CI: 0.996–0.998; and HR: 0.996, 95% CI: 0.995–0.998, respectively).

### 3.4. Dose Frequency Effects of ARB Usage on the Incidence of Dementia

Table 4 presents the dose frequency effect of ARBs on the incidence of dementia. All the groups demonstrated significant dose frequency effects compared with the group with an MPR of <20%. The group with an MPR of 70% to 80% demonstrated the most favorable protective effects (HR: 0.485, 95% CI: 0.375–0.626), followed by the group with an MPR of 60% to 70% (HR: 0.538, 95% CI: 0.420–0.689). The findings of the Cochran–Armitage test indicated a significant dose frequency effect of ARBs on dementia prevention (*p* = 0.005).

### 3.5. Dose Frequency Effects of ARB Usage on the Incidence of Dementia

**Figure 3:**
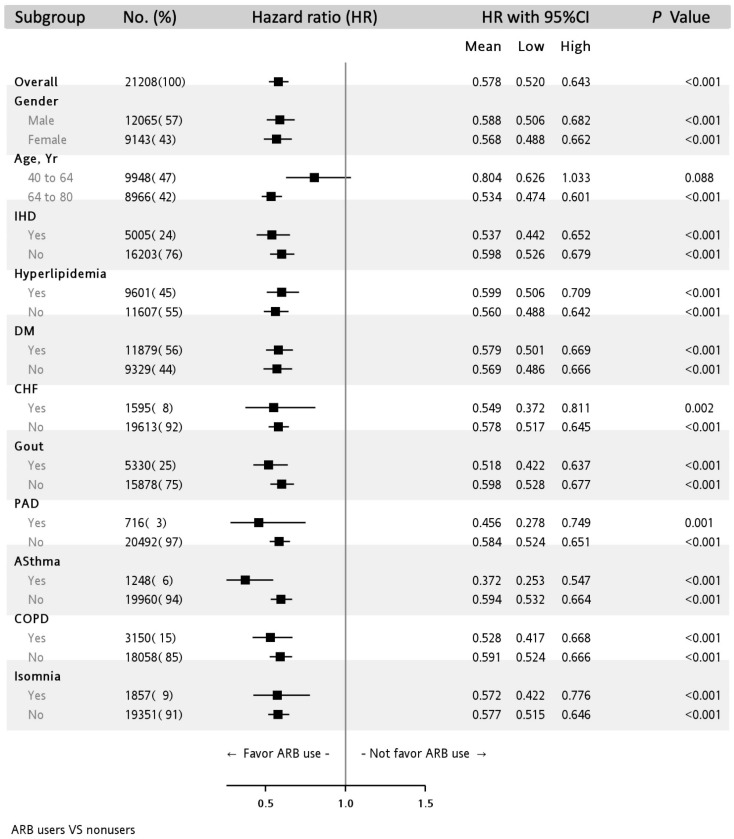
Subgroup analysis of the effect of ARB use on the incidence of dementia in the multivariable adjusting model.

Figure 3 depicts the association of dementia incidence with ARB usage stratified by covariates. The protective effect of ARB use on dementia was consistent and significant in all the subgroups except in the subgroup of the patients aged 40 to 64 years who demonstrated non-significant results (HR: 0.804, 95% CI: 0.626–1.033).

## 4. Discussion

In this nationwide study, we observed that ARBs exerted a significant protective effect on the incidence of dementia in our hypertensive CKD cohort. Such protective effect exhibited a dose frequency effect pattern. Moreover, the protective effect of ARBs on dementia was consistently observed in almost all the subgroups. Using real-world data, our study extends the current knowledge in the field by indicating that ARB use can prevent dementia in the hypertensive CKD population.

Several studies have determined the relationship between ARB usage and cognitive decline or dementia apart from its benefit in blood pressure reduction. Some studies have reported significantly positive findings. In the GEMS trial, Yasar et al conducted a post-hoc analysis and found that in individuals aged between 75 and 96 years, ARB usage more significantly reduced the incidence of Alzheimer disease dementia compared with no antihypertensive medication use (HR: 0.31, 95% CI: 0.14–0.68; *p* = 0.003) in the presence of normal baseline cognitive function [14]. Davies et al reported that in individuals aged over 60 years, an inverse association was observed between ARB usage and the development of Alzheimer disease dementia (odds ratio [OR]: 0.47, 95% CI: 0.37–0.58, *p* < 0.001) and vascular dementia (OR: 0.70, 95% CI: 0.57–0.85, *p* < 0.001) [15]. In their meta-analysis, Zhuang et al observed a lower incidence of Alzheimer’s disease (risk ratio: 0.69, 95% CI: 0.44–0.93) in ARB users [16]. Chiu et al reported that ARB users had a lower risk of both Alzheimer disease dementia (HR: 0.53, 95% CI: 0.43–0.64) and vascular dementia (HR: 0.63, 95% CI: 0.54–0.73) [17]. However, some studies have reported no significant association between ARB usage and dementia. In their meta-analysis, Ding et al did not observe an association of ARB use with incident Alzheimer’s disease (HR: 0.97; 95% CI: 0.82–1.15) and incident dementia (HR: 0.92, 95% CI: 0.75–1.13) in the high blood pressure stratum (systolic blood pressure ≥ 140 mmHg or diastolic blood pressure ≥ 90 mmHg) and in their counterparts with normal blood pressure (HR: 0.97, 95% CI: 0.8–1.18 and HR: 0.90, 95% CI: 0.69–1.19, respectively) [18]. Another meta-analysis by Peter et al demonstrated no association between the usage of ARBs and the risk of dementia in individuals aged >65 years (HR: 0.95, 95% CI: 0.56–1.61) [19]. 

Kuan et al found that among patients with diabetes mellitus and hypertension, all-cause dementia risk was lower in those receiving ARBs (HR: 0.60, 95% CI: 0.37–0.97) [24]. Other studies have reported no difference in the risk of dementia between ACEI and ARB users among patients with heart failure [25] or atrial fibrillation [26]. In their subgroup analysis (*n* = 5513), Chiu et al reported that ARB use could prevent dementia in Taiwanese patients with CKD (HR: 0.37, 95% CI: 0.30–0.46) [17]; this finding is consistent with that of our study. However, our study provided more robust evidence regarding the protective effect of ARBs on dementia in the CKD population.

Current guidelines recommend that ACEIs or ARBs should be used in patients with CKD who have high blood pressure [27,28] because of several benefits. ACEIs and ARBs have been shown to delay the progression of CKD [11,12], and ACEI or ARB use was associated with a lower risk of heart failure or death [11]. Because hypertension may cause cognitive decline through decreased cerebral perfusion or vascular remodeling [29], theoretically, the management of hypertension would reduce cognitive decline. In addition, a recent study suggested that ARBs exert neuroprotective effects independent of their hypotensive effects by modulating angiotensin receptors within the brain [13]. Although a definite consensus regarding the role of ARBs in the prevention of dementia is not available, our study suggests that a lower incidence of dementia may be an added benefit for patients with CKD who are already indicated to use ARBs. 

Apart from the effect of ARBs on dementia in the CKD population, we found that male sex was a protective factor for dementia; this finding is compatible with those of previous studies [30,31]. We observed that compared with ARB, ACEIs appeared to exert a weaker protective effect on dementia (HR: 0.897 vs. 0.578). This finding was supported by a recent randomized controlled trial indicating that candesartan resulted in superior neurocognitive outcomes compared with lisinopril [32]. A meta-analysis reported that diuretic antihypertensive drugs could reduce incident dementia risk [33]. However, our data showed that diuretic use was a risk factor for dementia (HR: 1.184, 95% CI: 1.083–1.296). The possible explanation for this finding is that our study population consisted of patients with CKD, and diuretic use in the CKD population indicates that they might have poor renal function, which is a risk factor for dementia [34]. Interestingly, 79.57% of ARB users were also users of ACEI. A possible explanation may be that these patients may having been using ACEI within the 1 year before the index rate and had later transitioned to ARB use.

The strengths of this study are as follows. First, because this was a nationwide population-based cohort study, its results can be generalized to the Chinese population. Second, our follow-up duration of 11 years was adequate to examine the primary outcomes. Moreover, we adjusted for some potential confounders; thus, the inference would be reliable. Finally, we minimized the overcoding of dementia by using the approved application for the catastrophic illness certificate of dementia in the NHI as our definition of dementia, and the diagnoses were made according to a strict protocol by neurologists or psychologists. Thus, the diagnoses of dementia were reliable in our study. Despite its strengths, our study still has some limitations. Information regarding potential confounding factors that are associated with dementia, including the body mass index, blood pressure, socioeconomic status, and lifestyle, was not available in the NHI database. Second, the severity of CKD stages could not be determined in the NHIRD, which might bias our inference. Finally, our study included only Taiwanese patients. Therefore, the results still need further verification before applying to other populations. 

## 5. Conclusions

Our study provides insights into the protective effects of ARB on dementia in the hypertensive CKD population in which ACEIs and ARBs are recommended for delaying CKD progression. Moreover, the protective effects of ARBs on dementia increased with an increase in the frequency of ARB use.

## Figures and Tables

**Table 1:** Baseline characteristics of chronic kidney disease (CKD) patients with hypertension.

	Total (*n* = 21,208)	ARB Users (*n* = 17,466)	ARB Nonusers (*n* = 3742)	*p*
**Sex**				<0.001
Male (%)	12,065 (43.11%)	9797 (56.09%)	2268 (60.61%)	
Female (%)	9143 (56.89%)	7669 (43.91%)	1474 (39.39%)	
**Age**	61.1 ± 12.1	60.8 ± 12	62.4 ± 12.7	<0.001
20–39 (%)	2294 (10.82%)	1894 (10.84%)	400 (10.69%)	<0.001
40–64 (%)	9948 (46.91%)	8412 (48.16%)	1536 (41.05%)	
65–80 (%)	8966 (42.28%)	7160 (40.99%)	1806 (48.26%)	
**Comorbidities**				
IHD (%)	5005 (23.6%)	4104 (23.5%)	901 (24.08%)	0.447
Hyperlipidemia (%)	9601 (45.27%)	8206 (46.98%)	1395 (37.28%)	<0.001
Diabetes mellitus (%)	11,879 (56.01%)	10,041 (57.49%)	1838 (49.12%)	<0.001
Atrial fibrillation (%)	362 (1.71%)	270 (1.55%)	92 (2.46%)	<0.001
CHF (%)	1595 (7.52%)	1260 (7.21%)	355 (8.95%	<0.001
Gout (%)	5330 (25.13%)	4358 (25.98%)	972 (24.95%)	0.190
PAD (%)	716 (3.38%)	580 (3.32%)	136 (3.63%)	0.335
Asthma (%)	1248 (5.88%)	204 (5.45%)	1044 (5.98%)	0.214
COPD (%)	3150 (14.85%)	2565 (14.69%)	585 (15.63%)	0.139
Major depression (%)	231 (1.09%)	183 (1.05%)	48 (1.28%)	0.208
Parkinson’s disease (%)	179 (1.09%)	125 (0.72%)	54 (1.44%)	<0.001
Rheumatoid arthritis (%)	252 (1.19%)	202 (1.16%)	50 (1.34%)	0.357
Insomnia (%)	1857 (8.76%)	1517 (8.69%)	340 (9.09%)	0.431
Thyrotoxicosis (%)	215 (1.01%)	173 (0.99%)	42 (1.12%)	0.464
Hypothyroidism (%)	175 (0.83%)	144 (0.82%)	31 (0.83%)	0.980
**Drugs**				
NSAIDs (%)	8947 (42.19%)	7350 (42.08%)	1597 (42.68%)	0.502
Acetaminophen (%)	8324 (39.25%)	6787 (38.86%)	1537 (41.07%)	0.011
Anti-platelet agents (%)	8504 (40.1%)	7080 (40.54%)	1424 (38.05%)	0.004
Anticoagulants (%)	329 (1.55%)	265 (1.52%)	64 (1.71%)	0.385
BZDs (%)	4795 (22.61%)	3846 (22.02%)	949 (25.36%)	<0.001
Insulin (%)	2204 (10.39%)	1869 (10.7%)	335 (8.95%)	0.001
Statin (%)	6587 (31.06%)	5743 (32.88%)	844 (22.55%)	<0.001
Metformin (%)	7340 (34.61%)	6338 (36.29%)	1002 (26.78%)	<0.001
ACEI (%)	5750 (27.11%)	4575 (79.57%)	1175 (20.43%)	<0.001
CCB (%)	11,762 (55.46%)	9951 (56.97%)	1811 (48.4%)	<0.001
Beta-blocker (%)	7970 (37.58%)	6629 (37.95%)	1341 (35.84%)	0.015
Diuretics (%)	7335 (34.59%)	6047 (34.62%)	1288 (34.42%)	0.814

Abbreviation: ARB, angiotensin receptor inhibitor; IHD, ischemic heart disease; CHF, chronic heart failure; PAD, peripheral artery disease, COPD, chronic obstructive pulmonary disease, NSAID, non-steroid anti-inflammatory drug; BZD, benzodiazepine; ACEI, angiotensin-converting enzyme inhibitor; CCB, calcium channel blocker.

**Table 2:** Risk for developing dementia by using Cox competing risk model.

	Crude		Multivariable	
	HR (95%CI)	*p*	aHR (95%CI)	*p*
ARB (vs. non)	0.645 (0.581–0.716)	<0.001	0.578 (0.520–0.643)	<0.001
Male (vs. female)	0.693 (0.638–0.754)	<0.001	0.823(0.753–0.899)	<0.001
Age				
20–39 (reference)	1		1	
40–64	21.440 (9.588–47.944)	<0.001	19.166 (8.56–42.876)	<0.001
65–80	111.6 (50.076–248.782)	<0.001	90.681 (40.645–202.316)	<0.001
**Comorbidities**				
Ischemic Heart Disease (vs. non)	1.735 (1.586–1.899)	<0.001	1.138 (1.031–1.256)	0.010
Hyperlipidemia (vs. non)	0.939 (0.864–1.021)	0.142	0.951 (0.864–1.047)	0.306
Diabetes mellitus (vs. non)	1.298 (1.192–1.413)	<0.001	1.172 (1.045–1.314)	0.006
Atrial fibrillation (vs. non)	2.508 (1.936–3.249)	<0.001	1.267 (0.966–1.662)	0.087
CHF (vs. non)	1.907 (1.647–2.209)	<0.001	1.180 (1.009–1.380)	0.038
Gout (vs. non)	1.040 (0.945–1.144)	0.421	1.024 (0.925–1.133)	0.648
PAD (vs. non)	1.715 (2.810–5.108)	<0.001	1.144 (0.938–1.395)	0.184
Asthma (vs. non)	1.594 (1.368–1.857)	<0.001	1.029 (0.852–1.243)	0.765
COPD (vs. non)	1.769 (1.596–1.961)	<0.001	1.218 (1.071–1.385)	0.002
Major depression (vs. non)	2.260 (1.703–3.000)	<0.001	2.065 (1.548–2.754)	<0.001
Parkinson’s disease (vs. non)	3.789 (2.810–5.108)	<0.001	1.717 (1.270–2.321)	<0.001
Rheumatoid arthritis (vs. non)	1.451 (1.034–2.037)	0.031	1.117 (0.794–1.572)	0.525
Insomnia (vs. non)	1.816 (1.607–2.052)	<0.001	1.334 (1.174–1.516)	<0.001
Thyrotoxicosis (vs. non)	1.153 (0.789–1.685)	0.463	1.109 (0.756–1.628)	0.595
Hypothyroidism (vs. non)	1.229 (0.800–1.899)	0.346	0.880 (0.570–1.358)	0.563
**Drugs**				
NSAIDs (vs. non)	1.272 (1.170–1.383)	<0.001	1.020 (0.931–1.118)	0.664
Acetaminophen (vs. non)	1.285 (1.181–1.398)	<0.001	1.010 (0.922–1.107)	0.831
Anti–platelet agents (vs. non)	1.637 (1.506–1.780)	<0.001	1.125 (1.028–1.231)	0.010
Anticoagulants (vs. non)	1.870 (1.383–2.530)	<0.001	1.235 (0.900–1.694)	0.190
BZDs (vs. non)	1.794 (1.640–1.962)	<0.001	1.279 (1.159–1.412)	<0.001
Insulin (vs. non)	1.224 (1.063–1.410)	<0.001	1.177 (1.013–1.366)	0.032
Statins (vs. non)	0.994 (0.909–1.088)	0.904	1.001 (0.902–1.111)	0.981
Metformin (vs non)	1.165 (1.069–1.27)	<0.001	1.126 (1.006–1.261)	0.039
ACEI (vs. non)	0.946 (0.86–1.041)	0.255	0.897 (0.813-0.989)	0.028
CCB (vs. non)	1.224 (1.125–1.332)	<0.001	1.016 (0.932–1.107)	0.726
Beta–blocker (vs. non)	0.998 (0.915–1.088)	0.957	0.918 (0.839–1.004)	0.060
Diuretic (vs. non)	1.508 (1.385–1.643)	<0.001	1.184 (1.083–1.296)	<0.001

Multivariable model: All the parameters were included in analysis; Abbreviation: ARB, angiotensin receptor inhibitor; CHF, chronic heart failure; PAD, peripheral artery disease, COPD, chronic obstructive pulmonary disease, NSAID, non-steroid anti-inflammatory drug; BZD, benzodiazepine; ACEI, angiotensin-converting enzyme inhibitor; CCB, calcium channel blocker.

**Table 3:** Competing risk analysis of ARB effects on dementia.

	ARB (Users vs. Nonusers)		Every Incremental of MPR of ARB	
	HR (95%CI)	*p* Value	aHR (95%CI)	*p* Value
Model 1	0.645 (0.581–0.716)	<0.001	0.997 (0.996–0.998)	<0.001
Model 2	0.607 (0.547–0.674)	<0.001	0.997 (0.996–0.998)	<0.001
Model 3	0.589 (0.530–0.655)	<0.001	0.997 (0.996–0.998)	<0.001
Model 4	0.578 (0.520–0.643)	<0.001	0.996 (0.995–0.998)	<0.001

Model 1 is a crude analysis. Multivariable model 2 is adjusted for age and gender. Multivariable model 3 comprises model 2 as well as adjustments for comorbidities, including ischemic heart disease, hyperlipidemia, diabetes, atrial fibrillation, congestive heart failure, gout, peripheral artery disease, asthma, chronic obstructive pulmonary disease, major depression, Parkinson’s disease, rheumatoid arthritis, insomnia, thyrotoxicosis, and hypothyroidism. Multivariable model 4 comprises model 3 as well as adjustments for medications of Non-steroid anti-inflammatory drugs, acetaminophen, anti–platelet agents, anticoagulants, benzodiazepines, Insulin, statins, metformin, angiotensin converting enzyme inhibitors, calcium channel blockers, beta–blockers and diuretics. Abbreviations: ARB, angiotensin receptor blocker; MPR, medication possession ratio; aHR, adjusted hazard ratio; CI, confidence interval.

**Table 4:** Dose frequency effect of ARB usage on dementia.

	Number	aHR * (95% CI)	*p* Value	Trend Test
MPR ≤ 20% (reference)	6322	1		*p* = 0.005
20% < MPR ≤ 30%	798	0.791 (0.632–0.990)	0.040	
30% < MPR ≤ 40%	801	0.718 (0.573–0.899)	0.003	
40% < MPR ≤ 50%	798	0.713 (0.566–0.899)	0.004	
50% < MPR ≤ 60%	860	0.728 (0.587–0.902)	0.003	
60% < MPR ≤ 70%	915	0.538 (0.420–0.689)	<0.001	
70% < MPR ≤ 80%	852	0.485 (0.375–0.626)	<0.001	
MPR > 80%	9862	0.704 (0.638–0.778)	<0.001	

* Multivariable adjusting model is as the model 4 in Table 3. Abbreviation: ARB, angiotensin receptor blocker; MPR, medication possession ratio; aHR, adjusted hazard ratio; CI, confidence interval.

## Data Availability

The present study used CKD thematic data from the NHIRD maintained by the Health and Welfare Data Science Center (HWDC), Ministry of Health and Welfare (MOHW), Taiwan.

## Supplementary Materials

The following are available online at https://www.mdpi.com/article/10.3390/jcm10215175/s1, Table S1: Tables for ICD codes used in defining CKD, Table S2: Tables for ICD codes used in defining baseline comorbidities. Table S3: Table for ATC codes used for defining usage of drugs.
