# Supplementary material for: Protective Effects of Angiotensin Receptor Blockers on the Incidence of Dementia in Patients with Chronic Kidney Disease: A Population-Based Nationwide Study
Source: J Clin Med. 2021 Nov 5;10(21):5175. doi: 10.3390/jcm10215175 (PMC8585022; doi:10.3390/jcm10215175)
Supplement: Supplementary file 1 [file jcm-10-05175-s001.zip › jcm-1448515-supplementary/supplementary/S1_CKD definition from NHIRD.pdf]

Table S1. Diagnosis Code for Chronic Kidney Disease in the thematic CKD cohort		
ICD9CM		Description
016.0		
	01600	Tuberculosis of kidney, unspecified
	01601	Tuberculosis of kidney, bacteriological or histological examination not done
	01602	Tuberculosis of kidney, bacteriological or histological examination unknown (at present)
	01603	Tuberculosis of kidney, tubercle bacilli found (in sputum) by microscopy
	01604	Tuberculosis of kidney, tubercle bacilli not found (in sputum) by microscopy, but found by bacterial culture
	01605	Tuberculosis of kidney, tubercle bacilli not found by bacteriological examination, but tuberculosis confirmed
	01606	Tuberculosis of kidney, tubercle bacilli not found by bacteriological or histological examination but tuberculosis confirmed by other methods [inoculation of animals]
095.4		
	0954	Syphilis of kidney
189		
	1890	Malignant neoplasm of kidney, except pelvis
	1891	Malignant neoplasm of renal pelvis
189.9		
	1899	Malignant neoplasm of urinary organ, site unspecified
223.0		
	2230	Benign neoplasm of kidney, except pelvis
236.91		
	23691	Neoplasm of uncertain behavior of kidney and ureter
250.4		
	25040	Diabetes with renal manifestations, Type II [non-insulin dependent type][NIDDM type][adult-onset type] or unspecified type ,not stated as uncontrolled
	25041	Diabetes with renal manifestations, Type I [insulin dependent type][IDDM][juvenile type], not stated as uncontrolled
	25042	Diabetes with renal manifestations, Type II [non-insulin dependent type][NIDDM type][adult-onset type] or unspecified type, uncontrolled
	25043	Diabetes with renal manifestations, Type I [insulin dependent type][IDDM][juvenile type], not stated as uncontrolled
255.1		
	2551	Hyperaldosteronism (Bartter's syndrome)
270.8		
	2708	Other specified disorders of amino-acid metabolism
271.0		
	2710	Glycogenosis

Table S1. Diagnosis Code for Chronic Kidney Disease in the thematic CKD cohort	
ICD9CM	Description
271.4	
271.4	Renal glycosuria
272.7	
2727	Lipidoses
274.1	
27410	Gouty nephropathy, unspecified
27411	Uric acid nephrolithiasis
27419	Other gouty nephropathy
283.11	
28311	Non-autoimmune hemolytic anemias, Hemolytic-uremic
403	
40300	Malignant hypertensive renal disease without mention of renal failure
40301	Malignant hypertensive renal disease with renal failure
40310	Benign hypertensive renal disease without mention of
40311	Benign hypertensive renal disease with renal failure
40390	Unspecified hypertensive renal disease without mention of renal failure
40391	Unspecified hypertensive renal disease with renal failure
404	
40400	Malignant hypertensive heart and renal disease without mention of congestive heart failure or renal failure
40401	Malignant hypertensive heart and renal disease with congestive heart failure
40402	Malignant hypertensive heart and renal disease with renal failure
40403	Malignant hypertensive heart and renal disease with congestive heart failure and renal failure
40410	Benign hypertensive heart and renal disease without mention of congestive heart failure or renal failure
40411	Benign hypertensive heart and renal disease with congestive heart failure
40412	Benign hypertensive heart and renal disease with renal failure
40413	Benign hypertensive heart and renal disease with congestive heart failure and renal failure
40490	Unspecified hypertensive heart and renal disease without mention of congestive heart failure or renal failure
40491	Unspecified hypertensive heart and renal disease with congestive heart failure
40492	Unspecified hypertensive heart and renal disease with renal failure
40493	Unspecified hypertensive heart and renal disease with congestive heart failure and renal failure

Table S1. Diagnosis Code for Chronic Kidney Disease in the thematic CKD cohort

ICD9CM		Description
440.1		
	4401	Atherosclerosis of renal artery
442.1		
	4421	Aneurysm of renal artery
447.3		
	4473	Hyperplasia of renal artery
572.4		
	5724	Hepatorenal syndrome
580		
	5800	Acute glomerulonephritis, with lesion of proliferative glomerulonephritis
	5804	Acute glomerulonephritis with lesion of rapidly progressive glomerulonephritis
	58081	Acute glomerulonephritis in diseases classified elsewhere
	58089	Acute glomerulonephritis with other specified pathological lesion in kidney
	5809	Acute glomerulonephritis with unspecified pathological lesion in kidney
581		
	5810	Nephrotic syndrome, with lesion of proliferative
	5811	Nephrotic syndrome, with lesion of membranous
	5812	Nephrotic syndrome, with lesion of membranous proliferative glomerulonephritis
	5813	Nephritic syndrome, with lesion of minimal change glomerulonephritis
	58181	Nephrotic syndrome in diseases classified elsewhere
	58189	Nephrotic syndrome, with other specified pathological lesion in kidney
	5819	Nephrotic syndrome with unspecified pathological lesion in kidney
582		
	5820	Chronic glomerulonephritis, with lesion of proliferative glomerulonephritis
	5821	Chronic glomerulonephritis, with lesion of membranous glomerulonephritis
	5822	Chronic glomerulonephritis, with lesion of membranous glomerulonephritis
	5823	Chronic glomerulonephritis, with lesion of membranous proliferative glomerulonephritis
	5824	Chronic glomerulonephritis, with lesion of rapidly progressive glomerulonephritis
	58281	Chronic glomerulonephritis in diseases classified elsewhere
	58289	Chronic glomerulonephritis, with other specified pathological lesion in kidney
	5829	Chronic glomerulonephritis with unspecified

Table S1. Diagnosis Code for Chronic Kidney Disease in the thematic CKD cohort		
ICD9CM		Description
		pathological lesion in kidney
583		
	5830	Nephritis and nephropathy, not specified as acute or chronic, with lesion of proliferative glomerulonephritis
	5831	Nephritis and nephropathy, not specified as acute or chronic, with lesion of membranous glomerulonephritis
	5832	Nephritis and nephropathy, not specified as acute or chronic, with lesion of membranous proliferative glomerulonephritis
	5834	Nephritis and nephropathy, not specified as acute or chronic, with lesion of rapidly progressive glomerulonephritis
	5836	Nephritis and nephropathy, not specified as acute or chronic, with lesion of renal cortical necrosis
	5837	Nephritis and nephropathy, not specified as acute or chronic, with lesion of renal medullary necrosis
	58381	Nephritis and nephropathy, not specified as acute or chronic, in diseases classified elsewhere
	58389	Nephritis and nephropathy, not specified as acute or chronic, with other specified pathological lesion in kidney
	5839	Nephritis and nephropathy, not specified as acute or chronic, with unspecified pathological lesion in kidney
584		
	5845	Acute renal failure, with lesion of tubular necrosis
	5846	Acute renal failure, with lesion of renal cortical necrosis
	5847	Acute renal failure, with lesion of renal medullary (papillary) necrosis
	5848	Acute renal failure, with other specified pathological lesion in kidney
	5849	Acute renal failure, unspecified
585		
	585	Chronic renal failure
586		
	586	Renal failure, unspecified
587		
	587	Renal sclerosis, unspecified
588		
	5880	Renal osteodystrophy
	5881	Nephrogenic diabetes insipidus
	5888	Other specified disorders resulting from impaired renal
	5889	Unspecified disorder resulting from impaired renal function
589		
	5891	Bilateral small kidneys of unknown cause
	5899	Small kidney of unknown cause, unspecified
591		

Table S1. Diagnosis Code for Chronic Kidney Disease in the thematic CKD cohort

ICD9CM	Description
591	Hydronephrosis
593	
5933	Stricture or kinking of ureter
5939	Unspecified disorder of kidney and ureter
599.6	
5996	Urinary obstruction, unspecified
642.1	
64210	Hypertension secondary to renal disease, complicating pregnancy, childbirth, and the puerperium, unspecified as to episode of care or not applicable
64211	Hypertension secondary to renal disease, complicating pregnancy, childbirth, and the puerperium, delivered, with or without mention of antepartum condition
64212	Hypertension secondary to renal disease, complicating pregnancy, childbirth, and the puerperium, delivered, with mention of postpartum complication
64213	Hypertension secondary to renal disease, complicating pregnancy, childbirth, and the puerperium, antepartum condition or complication
64214	Hypertension secondary to renal disease, complicating pregnancy, childbirth, and the puerperium, postpartum condition or complication
646.2	
64620	Unspecified renal disease in pregnancy, without mention of hypertension, unspecified as to episode of care or not
64621	Unspecified renal disease in pregnancy, without mention of hypertension, delivered, with or without mention of antepartum condition
64622	Unspecified renal disease in pregnancy, without mention of hypertension, delivered, with mention of postpartum
64623	Unspecified renal disease in pregnancy, without mention of hypertension, antepartum condition or complication
64624	Unspecified renal disease in pregnancy, without mention of hypertension, postpartum condition or complication
753	
7530	Renal agenesis and dysgenesis
75312	Polycystic kidney, unspecified type
75313	Polycystic kidney, autosomal dominant
75314	Polycystic kidney, autosomal recessive
75315	Renal dysplasia
75316	Medullary cystic kidney
75317	Medullary sponge kidney
75319	Other specified cystic kidney disease

Table S1. Diagnosis Code for Chronic Kidney Disease in the thematic CKD cohort	
ICD9CM	Description
753.2	
75320	Unspecified obstructive defect of renal pelvis and ureter
75321	Congenital obstruction of ureteropelvic junction
75322	Congenital obstruction of ureterovesical junction
75329	Other obstructive defect of renal pelvis and ureter
7533	Other specified anomalies of kidney
759.5	
7595	Tuberous sclerosis
794.4	
7944	Nonspecific abnormal results of function studies, kidney
984.9	
9849	Toxic effect of unspecified lead compound Lead
V-code	
V420	Kidney replaced by transplant
V451	Renal dialysis status
V594	Donors of kidney

Source: "Chronic Kidney Disease Prevention Technology Research Project" of National Health Administration, Ministry of Health and Welfare in Taiwan
