# Supplementary material for: Protective Effects of Angiotensin Receptor Blockers on the Incidence of Dementia in Patients with Chronic Kidney Disease: A Population-Based Nationwide Study
Source: J Clin Med. 2021 Nov 5;10(21):5175. doi: 10.3390/jcm10215175 (PMC8585022; doi:10.3390/jcm10215175)
Supplement: Supplementary file 1 [file jcm-10-05175-s001.zip › jcm-1448515-supplementary/supplementary/S2_Code for disease.pdf]

Table S2. Code for disease		
	ICD-9-CM	ICD-10-CM
Comorbidities		
Hypertension	401 – 405	I10-15
Diabetes mellitus	250	E08-E13
Hyperlipidemia	272.0–272.4	E78
Ischemic Heart Disease	410 – 414	I20-I25
CHF	428.0, 428.1, 428.9	I50
Stroke	430–438	I60-I69
PAOD	440.2-9, 443.9	I70.2-I70.9
COPD	491, 496	J44
Cancer	140-208	C00.0-C96.9
Atrial fibrillation	427.31	I48
Asthma	493	J45
Major Depression Dis-order	296.2-3	F32-F33
Parkinson's Disease	332	G20-G21
Rheumatoid Arthritis	714.0, 714.1, 714.2, 714.81	M05-M06
Thyrotoxicosis	240	E05
Hypothyroidism	244, 245.2	E01.8, E02-E03
Insomnia	780.51, 780.52	G47.0, F51.0
Gout	274	M10
Clinical outcomes		
Dementia (eligible for Severe Illness for National Health Insurance Administration IC Card)	290	F01.50, F01.51, F03.90, F03.91

Abbreviation:
ICD, international classification of disease; CHF, Congestive heart failure; PAOD, Peripheral artery occlusion disease; COPD, Chronic obstructive pulmonary disease.
