# Supplementary material for: Protective Effects of Angiotensin Receptor Blockers on the Incidence of Dementia in Patients with Chronic Kidney Disease: A Population-Based Nationwide Study
Source: J Clin Med. 2021 Nov 5;10(21):5175. doi: 10.3390/jcm10215175 (PMC8585022; doi:10.3390/jcm10215175)
Supplement: Supplementary file 1 [file jcm-10-05175-s001.zip › jcm-1448515-supplementary/supplementary/S3_Code for medication.pdf]

Table S3.Code for drugs		
Drug type	ATC classification system codes	Drug name
ARB	C09C, C09D	Losartan, Eprosartan, Valsartan, Irbesartan, Candesartan, Telmisartan, Olmesartan, Azilsartan
NSAID	M01AB, M01AC, M01AE, M01AG, M01AH, M01AX01	Indomethacin, Sulindac, Tolmetin, Diclofenac, Alclofenac, Etodolac, Acemetacin, Ketorolac, Aceclofenac, Piroxicam, Tenoxicam, Meloxicam, Ibuprofen, Naproxen, Ketoprofen, Fenoprofen, Fenbufen, Flurbiprofen, Tiaprofenic Acid, Alminoprofen, Naproxen, Mefenamic Acid,,Tolfenamic Acid Flufenamic Acid, Meclofenamic Acid, Celecoxib, Rofecoxib, Etoricoxib, Nabumetone, Niflumic Acid
Acetaminophen	M03BA52, M03BB03, M03BB52, M03BB53, M03BC01, M03BC51, N02AC, N02AX52, N02BA57, N02BE, R05X	Acetaminophen
Anti-platelet agents	B01AC, M01BA03, N02AB, N02BA01, N02BA51	Clopidogrel, Ticlopidine, Acetylsalicylic Acid, Dipyridamole, Epoprostenol, Iloprost, Abciximab, Eptifibatide, Tirofiban, Treprostinil, Prasugrel, Cilostazol, Ticagrelor, Selexipag,
Anticoagulants	B01AA, B01AB	Phenindione, Warfarin, Heparin, Dalteparin, Enoxaparin, Nadroparine, Tinzaparin
Benzodiazepines	N05BA, N05CD	Diazepam, Chlordiazepoxide, Medazepam, Oxazepam, Clorazepate, Lorazepam, Bromazepam, Clobazam, Prazepam, Alprazolam, Nordazepam, Fludiazepam, Cloxazolam, Oxazolam, Flurazepam, Nitrazepam, Flunitrazepam, Estazolam, Triazolam, Lormetazepam, Temazepam, Midazolam, Brotizolam, Nimetazepam
Insulin	A10A	Insulin
Statin	C10AA	Simvastatin, Lovastatin, Pravastatin, Fluvastatin, Atorvastatin, Rosuvastatin, Pitavastatin
Metformin	A10BA02, A10BD, A10BD02, A10BD03, A10BD05, A10BD07, A10BD08, A10BD10, A10BD11, A10BD13, A10BD15, A10BD20	Metformin

ACEI	C09A, C09B	Captopril, Enalapril, Lisinopril, Perindopril, Ramipril, Quinapril, Benazepril, Cilazapril, Fosinopril, Imidapril
Beta blocking agents	C07A, C07B, C07CA03, C07DA06	Alprenolol, Oxprenolol, Pindolol, Propranolol, Timolol, Sotalol, Nadolol, Carteolol, Bupranolol, Metoprolol, Atenolol, Acebutolol, Betaxolol, Bevantolol, Bisoprolol, Esmolol, Nebivolol, Labetalol, Carvedilol
CCB	C08C, C08D, C09BB, C09DB, C09DX01, C09DX03 C09XA, C10BX	Amlopidine, Felodipine, Isradipine, Nicardipine, Nifedipine, Nimodipine, Nisoldipine, Nitrendipine, Lacidipine, Barnidipine, Lercanidipine, Cilnidipine, Benidipine, Verapamil, Diltiazem
Diuretic	C03A, C03B, C03C, C03D, C03E, B05BC9	Bendroflumethiazide, Hydrochlorothiazide, Trichlormethiazide, Cyclopenthiazide, Benzylhydrochlorothiazide, Chlorthalidone, Metolazone, Indapamide, Furosemide, Bumetanide Torsemide, Ethacrynic Acid, Potassium Canrenoate, Spironolactone, Eplerenone, Amiloride, Triamterene,

Abbreviation:
ARB, Angiotensin receptor blockers; NSAID, Non-Steroidal Anti-Inflammatory Drug; ACEI, Angiotensin-converting enzyme inhibitors; CCB, Calcium channel blockers
